# Supplementary material for: Assisting students’ writing with computer-based concept map feedback: A validation study of the CohViz feedback system
Source: PLoS One. 2020 Jun 29;15(6):e0235209. doi: 10.1371/journal.pone.0235209 (PMC7323980; doi:10.1371/journal.pone.0235209)
Supplement: Attachment — Submitted filename: PLoS One.docx [file pone.0235209.s001.docx]

PLoS One

**Peer review**

Assisting students’ writing with computer-based concept map feedback: A validation study of

the CohViz feedback system

**Major comments:**

This is a helpful paper because it validates the use of a specific tool for student feedback on their writing. It is unclear how the authors think this tool is being used and how it can be used.

The paper is very long and some information seems unnecessary. There are, for example, many examples. There is a lot of repetition both on a sentence level and in terms of information repeated again in later explanations that should be adjusted.

The paper is flawed as a piece of writing because of the way that the two studies were basically pasted together as well as the chronological presentation of information. The paper needs to be reorganized to explain that the authors studied validity and reliability of the CohViz system and have one methods section that outlines all the methods (preplanned and post hoc) and one results and discussion section. The paper does need to be rewritten and some of the framing and examples have to be sacrificed to make the paper work as a whole.

The current overall discussion section is very good—really concise, helpful, and well-written. The rest of the paper should follow this model and state things once and without so much explanation.

The results should be tabulated and not embedded in the text.

**Specific comments:**

**Abstract:**

- Overall this is clear and well written, describing a helpful pair of studies about a tool already in use.
- It would be helpful to know the discipline of the teachers using CohViz
- The authors should be careful not to use the same word multiple times in a sentence or phrase
- It would be helpful to know the general length of the texts assessed

**Introduction:**

- Please delete the information about aspirin and the following sentence. Your prior point is cogent and adequate and does not need more support.
- It would be helpful to have a little more information about the setting for the studies. Just a couple of words—college? High school? English? Writing? Wikipedia?
- Overall—the introduction does not seem to me to be introducing the major concepts that occur later in the paper and therefore it is confusing

**Computer-Based Systems on Students’ Cohesive Writing**

- I’m not sure what this section adds. Would it be sufficient to have a single paragraph providing a short overview of the idea of using a computer-based system for evaluating cohesion in the earlier section?

**CohViz: A Feedback System to Support Students’ Cohesive Writing**

- I still would like more information about the setting for using this tool—who uses it?
- I am unsure when and where the cited studies by Lachner and colleagues were completed. In a college? Recently? Why?

**Accuracy as a Critical Component of Effective Computer-Based Feedback, Reliability, Validity**

- I’m not sure what these sections add. Ideally, the section about CohViz should simply state the limitations of the prior studies, the actual needs of users, and the need for the current studies much more concisely, following the style of the current overall discussion.

**Overview of the Present Studies**

- I would not describe the studies as “empirical”
- I would describe the studies in terms of what they are testing (“reliability” and “validity”) instead of calling them Study 1 and Study 2 and expecting the reader to keep track of the shifts

**Study 1 Methods:**

- I find this section confusing, largely because I’m not sure who is doing what where and when and to which corpus of texts. Ideally the methods would identify in order:
  - The goal of the study (assess reliability of concept maps between machine and human coders)
  - How human coders were identified
  - Which corpus of work was used for CohViz versus human coders. How was the full corpus generated and in which setting and by whom? How was the smaller representative sample generated in which setting and why whom?
  - How were the outputs generated? Who reviewed them?
- I would move the explanation about how CohViz works to the introduction
- I would strongly recommend avoiding the use of “Lincoln freed the slaves” as an example. It would be highly offensive to many US readers. Please choose something more neutral. Maybe “Hollywood baked the rolls”?

**Study 1: Results and discussion:**

- Please move the statistical methods back to the methods section
- This discussion is far too long and has far too many little asides and explanations As a reader, it is helpful to have the results presented as:
  - How big were the samples reviewed? Did the representative sample for human coders adequately mimic the overall sample? How did you know that?
  - What actually was completed? How many concept maps were generated and reviewed?
  - How well did the two methods correlate?

**Study 2:**

- This seems very long and not clear because information that should be in the introduction is interspersed throughout the text, there are methods in the results section and too much explanation as the results are presented. It simply feels very similar to the problems I noted in Study 1, where the methods and results are not clearly laid out in a logical order, but rather seem to demand that the reader carefully track with the researchers.

**Overall discussion:**

This is generally well written and concise. Please describe the studies in terms of what they tested and not by numbers. It makes the work very hard to read.

**Limitations and future directions:**

This seems very wordy and a bit too speculative. I applaud the correctness of the limitations identified, but the lengthy caveats and explanations are not really helpful. More helpful are the practical modes of moving forward.
