# Supplementary material for: Assisting students’ writing with computer-based concept map feedback: A validation study of the CohViz feedback system
Source: PLoS One. 2020 Jun 29;15(6):e0235209. doi: 10.1371/journal.pone.0235209 (PMC7323980; doi:10.1371/journal.pone.0235209)
Supplement: Attachment — Submitted filename: Response to Reviewers.docx [file pone.0235209.s003.docx]

**Reviewer #1:**

**The paper as initially submitted was already a valuable addition to the literature and based on a well-designed study. Therefore the prior comments largely had to do with how the information was presented as well as the completeness of small details that are necessary to understand how the study was designed. This is an excellent writing revision, following my recommendations very well while also addressing the concerns of reviewer two.**

**More importantly, this rewrite made it possible to see that the statistics are well-done. It is evident that the rigor is adequate to answer the researchers' questions. This paper now provides more adequate information to help readers and researchers consider how they might use this technology in various academic settings.**

Thank you very much. We appreciate your feedback.

**Reviewer #2:**

**(No Response)**

**Reviewer #3:**

**1. Language problems: not detected**

**2. Presentation problems:
2.1 The functioning of the CohViz system is mainly textual. It would be good to present mathematical formulas used to calculate important measures of the text cohesion features used by the CohViz.**

Thank you for the suggestion. Under the sub-chapter *Structural indicators of the concept maps* we have now added a formal definition of the three central structural indicators of CohViz using graph theory notation (p. 15). We now describe the fragments, relations, and concepts in the language of graph theory and explain these definitions using the concept map in Figure 3 as an example. At the end of the sub-chapter we also refer to the public repository under which the algorithms (written in Python) for calculating these indicators can be found. Besides, we have added and described formulas that explain the calculation of the measures of *adjacent argument overlap* and *text level overlap* (p. 16-17).

**3. Other problems:
3.1 The link to data given by the Authors is invalid. Authors write: "All files are available on github at the following address: https://github.com/ch-bu/dataassisting-students-writing-with-computer-based-concept-map-feedback". This link goes to "Error 404 - page not found"**

We apologize for any inconvenience caused by the incorrect link. We have now uploaded the data and the analyses of the data to a public repository under the following address: [**https://doi.org/10.17605/OSF.IO/UHPF3**](https://doi.org/10.17605/OSF.IO/UHPF3) on the OSF portal. We have also published the algorithms we used to calculate the structural indicators of CohViz. The files for these calculations can be found in the *analysis/stuctural_indicators* directory of the repository. Also, a reference to the repository can be found in the manuscript on page 15.

**3.2 It would be good to give direct information who is the author of CohViz system. In the actual form of the text it is still unclear without reading the text of given references (e.g. [15]). Please consider the following change: CohViz is a graphical feedback which automatically provides students with concept maps as external representations to improve the cohesion of their writing (see Fig 1) [15,20,21]. => CohViz is a graphical feedback tool we developed, which automatically provides students with concept maps as external representations to improve the cohesion of their writing (see Fig 1) [15,20,21].**

We have incorporated the reviewer's suggestion and now report that we are the authors of the system (see the revised manuscript, p. 4).

**3.3 The main focus of the manuscript is the CohViz system developed by the Authors. Manuscript includes description of the system and validation of its reliability and accuracy. In such case PLOS One Software availability criteria should be met:

"PLOS ONE will consider submissions that present new methods, software, databases, or tools as the primary focus of the manuscript if they meet the following criteria:
(...) Validation: Submissions presenting methods, software, databases, or tools must demonstrate that the new tool achieves its intended purpose. (...) Availability: If the manuscript’s primary purpose is the description of new software or a new software package, this software must be open source, deposited in an appropriate archive, and conform to the Open Source Definition. (...) Please see:**
[**https://journals.plos.org/plosone/s/submission-guidelines#loc-methods-software-databases-and-tools**](https://journals.plos.org/plosone/s/submission-guidelines#loc-methods-software-databases-and-tools)**. Validation is presented. Availability is not met.**

In fact, we did not include a reference to the CohViz system in the manuscript. Accordingly, on page 4 of the revised manuscript, we have now included the reference to the system, which has been uploaded to the OSF public repository under the following address: <https://doi.org/10.17605/OSF.IO/SA53E>. We chose to publish the system under the MIT license as it conforms to the Open Source Definition (<https://opensource.org/licenses>) recommended by PLOS One. Under the MIT license, the software is freely available and may be freely distributed and modified.

**3.4 Authors write:

"In the validity study, we demonstrated the convergent and divergent validity of the CohViz system in comparison to measures of other well-established research tools for writing quality [35,43]"

Can Authors point out where in their manuscript the "measures of other well-established research tools for writing quality" are presented and compared with results for CohViz? Any comparison of values of selected measures for CohViz and considered "other" tools? Any table or figure?**

Indeed, the sentence was written misleadingly. By "other" we referred to the measures we reported in the chapter on *convergent and divergent measures of text cohesion*, which are reported in Table 2. We have revised the sentence, removed the word “other” and now report that we have used convergent and divergent measures from well-established assessment systems (see p. 27). Besides, we have added a box to Figure 2, in which we explain the processing of the Wikipedia corpus, and explicitly refer to the assessment system used by Hancke et al. During the revision we also noticed that we have used two terms to describe these systems in the manuscript: *computer-linguistic frameworks* and *assessment systems*. We now speak uniformly of *assessment systems*. **Summary: The language is good. The presentation should be improved. Data availability and software availability are not met. But what is most important area that needs fixing includes formal definitions of analyzed measures and comparison with other similar tools.

Recommendation: major rework**
